# Evaluating the Accuracy and Educational Potential of Generative AI Models in Pharmacy Education: A Comparative Analysis of ChatGPT and Gemini Across Bloom’s Taxonomy

**DOI:** 10.3390/pharmacy14010001

**Published:** 2025-12-23

**Authors:** Tuan Tran, Uyen Le, Victor Phan

**Affiliations:** College of Pharmacy, California Northstate University, 9700 West Taron Drive, Elk Grove, CA 95757, USA; tuan.tran@cnsu.edu (T.T.); uyen.le@cnsu.edu (U.L.)

**Keywords:** generative AI models, pharmacy education, Bloom’s Taxonomy, AI-assisted learning

## Abstract

This study evaluated the accuracy and educational potential of three generative AI models, ChatGPT 3.5, ChatGPT 4o, and Gemini 2.5, by addressing pharmacy-related content across three key areas: biostatistics, pharmaceutical calculations, and therapeutics. A total of 120 exam-style questions, categorized by Bloom’s Taxonomy levels (Remember, Understand, Apply, and Analyze), were administered to each model. Overall, the AI models achieved a combined accuracy rate of 77.5%, with ChatGPT 4o consistently outperforming ChatGPT 3.5 and Gemini 2.5. The highest accuracy was observed in therapeutics (83.3%), followed by biostatistics (81.7%) and calculations (67.5%). Performance was strongest at lower Bloom levels, reflecting proficiency in recall and conceptual understanding, but declined at higher levels requiring analytical reasoning. These findings suggest that generative AI tools can serve as effective supplementary aids for pharmacy education, particularly for conceptual learning and review. However, their limitations in quantitative and higher-order reasoning highlight the need for guided use and faculty oversight. Future research should expand to additional subject areas and assess longitudinal learning outcomes to better understand AI’s role in improving critical thinking and professional competence among pharmacy students.

## 1. Introduction

The rapid expansion of generative artificial intelligence (AI) has reshaped how knowledge is accessed, synthesized, and applied in higher education. Advanced language models such as OpenAI’s ChatGPT [1], Google’s Gemini DeepMind [2], and Anthropic’s Claude [3] have demonstrated remarkable capabilities in generating coherent, contextually relevant responses, fostering interactive learning, and supporting academic inquiry across disciplines. With the increasing accessibility of these AI tools, educators and students are exploring their potential to enhance learning experience and conceptual understanding. However, while their popularity grows, users may not be fully aware of their limitations, especially in specialized and high-stakes fields like pharmacy, where precision, analytical reasoning, and clinical judgment are essential. Understanding the actual performance and reliability of generative AI tools in this context is therefore critical to ensure their safe and effective integration into professional education.

Several studies have begun evaluating the educational applications of AI models within medical and health sciences. Early research demonstrated that ChatGPT performs competitively on standardized medical assessments such as the United States Medical Licensing Examination (USMLE), achieving scores comparable to or exceeding passing thresholds [4,5]. A recent systematic review and meta-analysis further confirmed that ChatGPT and similar large language models generate responses of generally acceptable accuracy and coherence in medical contexts, though variability persists across topics and question complexities [6]. Despite these advances, scholars have cautioned that current AI models may exhibit reasoning gaps, biases, or inconsistencies when presented with complex or quantitative problems, underscoring the need for further empirical validation [7].

Large Language Models (LLMs), such as those based on the transformer architecture, function primarily through a mechanism of “next-token prediction.” Rather than accessing a structured database of facts or performing deterministic calculations, these models generate responses by predicting the most likely subsequent word or symbol based on patterns learned from large datasets of text [8]. While this architecture allows for remarkable proficiency in natural language tasks, it presents inherent limitations in tasks requiring strict logic or arithmetic reasoning. In these contexts, the probabilistic nature of the model can lead to plausible-sounding but factually incorrect outputs. This phenomenon is widely called “hallucination” [9]. Recent evaluations in pharmacy education highlight this dichotomy as well. While models often perform at or near human levels on knowledge recall questions, their accuracy significantly declines on tasks involving multi-step pharmaceutical calculations, chemical structural analysis, or complex clinical reasoning. LLMs usually struggle to maintain logical consistency without specific prompting strategies or external tools [10].

Within pharmacy education, emerging evidence highlights the potential benefits and challenges of using AI tools to support learning and clinical reasoning. Carou-Senra et al. [11] reported that ChatGPT could assist pharmacy students in developing therapeutic decision-making skills by explaining pharmacologic concepts and evaluating treatment alternatives. Similarly, Goldman et al. [12] examined AI use during Advanced Pharmacy Practice Experiences (APPEs), finding that ChatGPT improved accessibility to drug information and supported Entrustable Professional Activities (EPAs), though its reliability varied by task type. Together, these studies suggest that AI models may serve as valuable educational supplements, particularly for conceptual understanding and knowledge retrieval, while human oversight remains necessary for verifying accuracy and contextual appropriateness.

Building on this growing body of literature, this study systematically evaluates the performance of three state-of-the-art generative AI models, ChatGPT 3.5, ChatGPT 4o, and Gemini 2.5, across core domains of the pharmacy curriculum: biostatistics, pharmaceutical calculations, and therapeutics. Using 120 exam-style questions mapped to Bloom’s Taxonomy levels (Remember, Understand, Apply, and Analyze), this research assesses each model’s accuracy across cognitive and topical dimensions. By comparing their outputs, the study aims to determine the extent to which AI tools can effectively support pharmacy education and identify their limitations in higher-order reasoning and quantitative problem-solving.

## 2. Materials and Methods

We administered 120 exam questions to ChatGPT 3.5, 4o, and Gemini 2.5. The questions were created by the domain expert faculty members based on widely used materials, including the RxPrep Course Book, APhA Complete Review for Pharmacy, Board Vitals, and UWorld for Pharmacy. The questions covered three main knowledge domains, including biostatistics, calculations, and therapeutics. Each topic area had 40 questions evenly distributed across 4 levels of Bloom’s Taxonomy (i.e., 10 questions per level), including Remember, Understand, Apply, and Analyze. The difficulty level of each question in the question pool was first determined by the domain expert faculty, then reviewed by two other faculty members. For conceptual questions, a multiple-choice or multiple-selection format was used with four answer choices. On the other hand, for calculation questions, the format of fill-in-the-blank was used. Initially, we administered these exam questions to ChatGPT 3.5 (February 2024) and evaluated its accuracy. After that, when the newer model ChatGPT 4o (May–June 2024) and Gemini 2.5 (July–August 2025) were available, we input the same questions and re-evaluated the accuracy. In our experiment, we selected the opt-out option in Data Controls in ChatGPT so that the previously entered data would not be used to improve the newer model; thus, the comparison across the models is valid and accurate. Generative AI was prompted to select the correct option listed in each question or to provide the correct answer for calculations. Questions were presented to each model one at a time during the study period, from February 2024 to August 2025. In our study, we used two different prompts for multiple-choice and calculation fill-in-the-blank questions, respectively.

- Prompt for multiple-choice questions: “Act as a clinical pharmacist using evidence-based pharmacy resources and guidelines (e.g., IDSA, ACC/AHA, ADA, KDIGO, Lexicomp, pharmacokinetics, drug-interaction data, etc.), analyze the multiple-choice question below, identifying the core therapeutic problem, and evaluating each option (A–D). Explain why the correct option is guideline-preferred and provide your response in the format: Reasoning → Why an option is correct/incorrect → FINAL ANSWER: [Letter—Option Text].” This prompt reflects how pharmacy students are trained to approach clinical questions by using evidence-based guidelines, pharmacokinetics, and drug interaction principles to evaluate clinical cases. In addition, our prompt also ensures that the AI models use credible pharmacy resources to justify their selection. The AI models’ instructed reasoning is intended to provide clear, clinically defensible decision-making, similar to NAPLEX and therapeutics exams.
- Prompt for Calculation fill-in-the-blank questions: “Act as a clinical pharmacist using validated pharmacy references for pharmacokinetic equations, dosing calculations, and clinical guidelines. Summarize the clinical data relevant to the calculation, then show clear step-by-step math using proper formulas, units, and assumptions. Apply pharmacy-standard PK and dosing principles to arrive at one definitive value and present your response as: Reasoning/steps → FINAL ANSWER: [Numeric Result + Units].” The designed prompt is to ensure that pharmacokinetic and dosing calculations are performed using validated pharmacy references, proper formulas, and clinically accurate assumptions. The required step-by-step structure reflects pharmacy program expectations. A single, precise final answer is generated consistently with professional practice.

The faculty members then evaluated and discussed the responses from the generative AI models to determine their correctness. In our study, the responses were classified into “Correct”, “Incorrect”, and “Partially Correct”. The detailed description of each category, along with examples of the exam questions, is provided in Table 1.

## 3. Results

### 3.1. Overall Accuracy Performance

**Figure 1:**
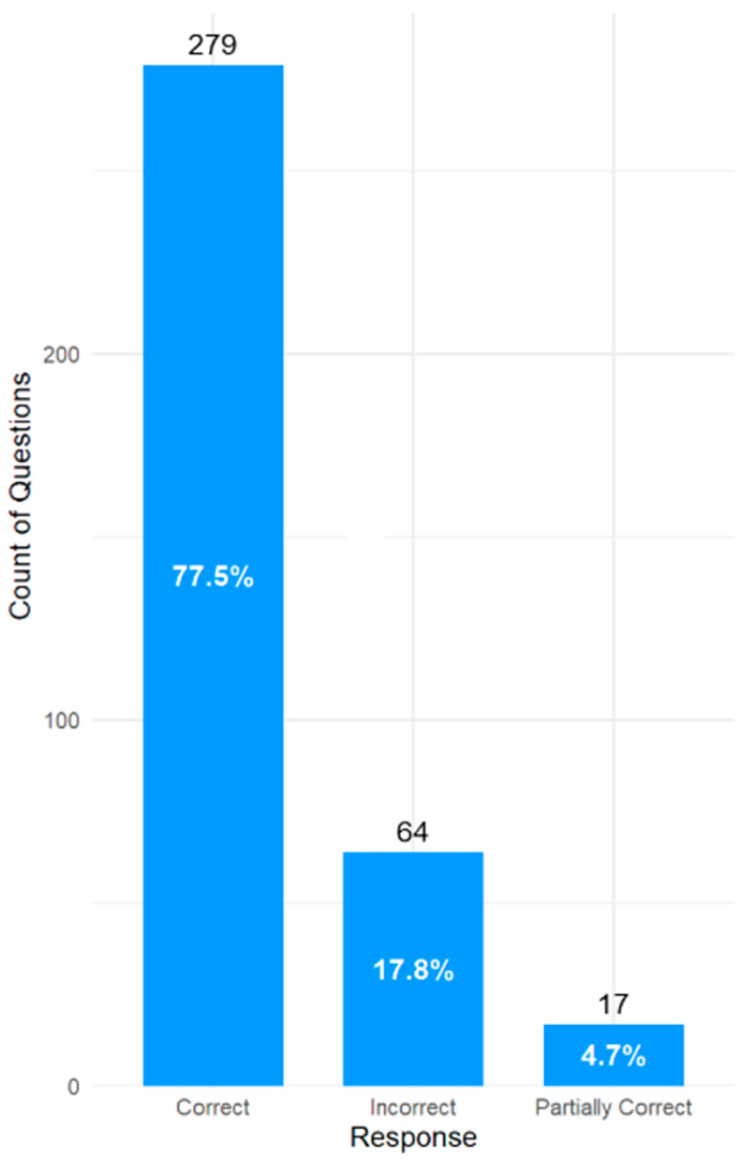
Overall accuracy of the responses for all models with 120 input questions.

Across all three generative AI models, a total of 360 questions were evaluated. This is because the original set of 120 questions was duplicated and given to each model separately. Out of the questions presented to all three models, there were 279 (77.5%) correct responses, 64 (17.8%) were incorrect, and 17 (4.7%) were partially correct. The assessment outcome across all three AI models based on the accuracy of the responses is shown in Figure 1.

### 3.2. Accuracy Performance by Topics

**Figure 2:**
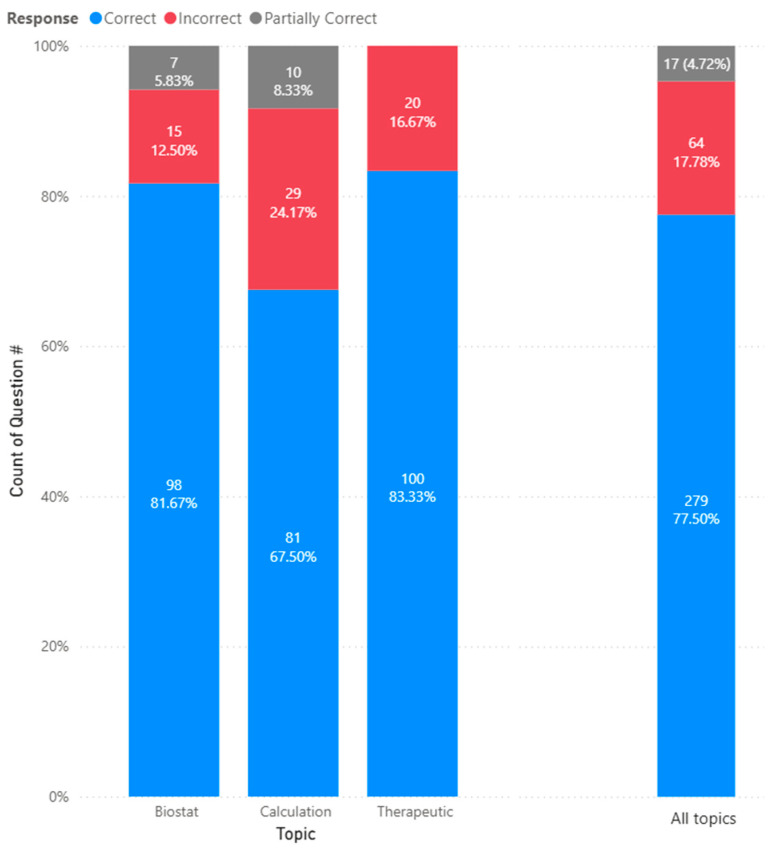
Generative response accuracy by content topics.

Next, we analyze the accuracy of the generative AI models’ responses by pharmacy topics. For therapeutics, across all three models, 100 (83.3%) were correct, and 20 (16.7%) were incorrect. For biostatistics, 98 (81.7%) were correct, 15 (12.50%) were incorrect, and 7 (5.8%) were partially correct. And finally, for calculations, 81 (67.5%) were correct, 29 (24.2%) were incorrect, and 10 (8.3%) were partially correct. Figure 2 shows the distribution of the accuracy of the responses by topics.

### 3.3. Performance by Topic and Model

**Figure 3:**
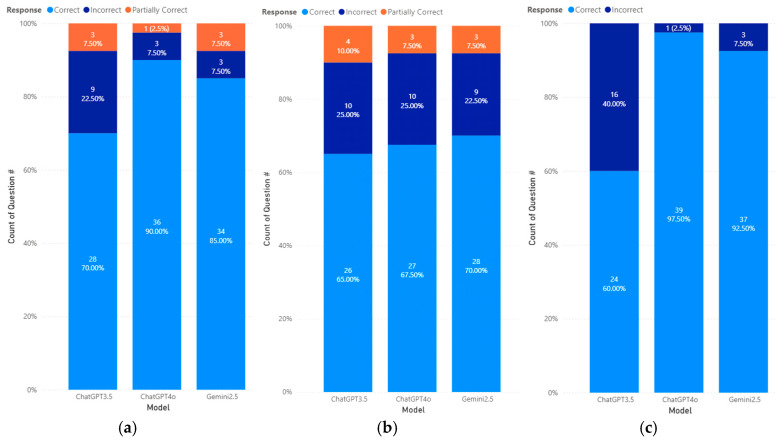
Accuracy performance of different models by topics: (**a**) Biostatistics, (**b**) Calculation, (**c**) Therapeutic.

Next, we analyze the performance of different models by topic, as shown in Figure 3. For the biostatistics topic, ChatGPT 3.5 answered 28 (70.0%) questions correctly, 3 (7.5%) questions partially correctly, and 9 (22.5%) questions incorrectly. On the other hand, ChatGPT 4o answered 36 (90.0%) questions correctly, with 3 (7.5%) questions partially correct and 1 (2.5%) question incorrect. Finally, Gemini 2.5 answered 34 (85.0%) questions correctly, with 3 (7.5%) questions partially correct and 3 (7.5%) questions incorrect.

For the calculation topic, ChatGPT 3.5 answered 26 (65.0%) questions correctly, with 4 (10.0%) questions partially correct and 10 (25.0%) questions incorrect. ChatGPT 4o answered 27 (67.5%) questions correctly, with 3 (7.5%) questions partially correct and 10 (25.0%) questions incorrect, and Gemini 2.5 answered 28 (70.0%) questions correctly, with 3 (7.5%) questions partially correct and 9 (22.5%) questions incorrect.

Finally, for the therapeutics topic, ChatGPT 3.5 answered 24 (60.0%) questions correctly and 16 (40%) questions incorrectly. With improvement, ChatGPT 4o answered 39 (97.5%) questions correctly and 1 (2.5%) incorrectly. Similarly, Gemini 2.5 answered 37 (92.5%) questions correctly and 3 (7.5%) questions incorrectly.

### 3.4. Accuracy Performance by Bloom’s Taxonomy

**Figure 4:**
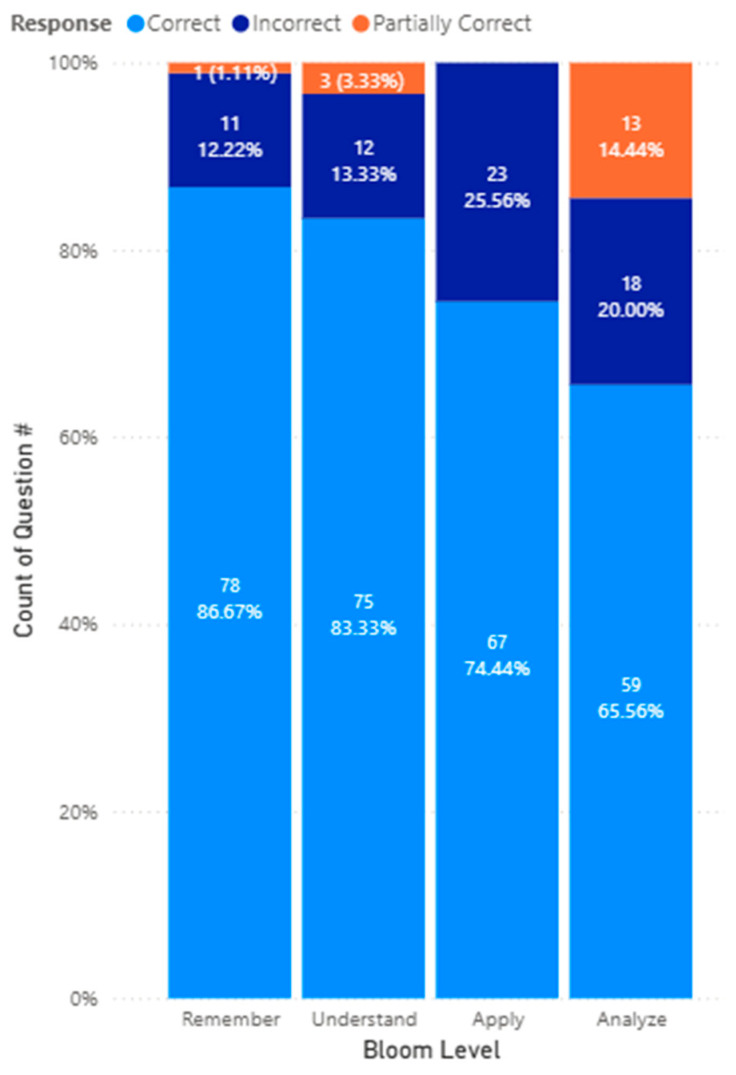
The accuracy performance of all three models by Bloom’s Taxonomy.

Across all three generative AI models, by Bloom’s Taxonomy, for questions categorized as Remember, 78 (86.7%) responses were correct, 1 (1.11%) response was partially correct, and 11 (12.22%) responses were incorrect. On the other hand, for questions categorized as Understand, 75 (83.3%) responses were correct, 3 (3.3%) responses were partially correct, and 12 (13.3%) responses were incorrect. For questions categorized as Apply, 67 (74.4%) responses were correct, and 23 (25.6%) responses were incorrect. Finally, for questions categorized as Analyze, 59 (65.6%) responses were correct, 13 (14.4%) responses were partially correct, and 18 (20.0%) responses were incorrect. The results are illustrated in Figure 4.

**Figure 5:**
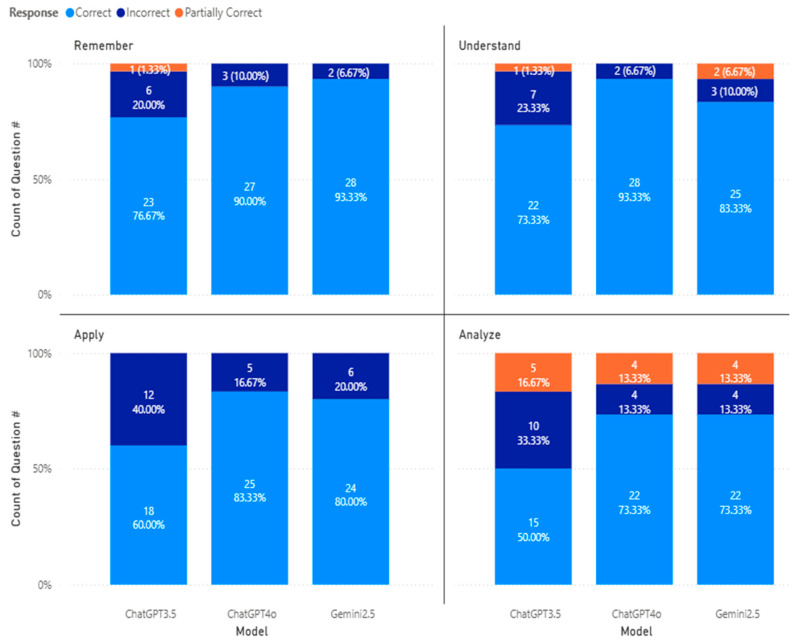
Accuracy performance of different models by Bloom’s Taxonomy levels.

Next, Figure 5 shows the accuracy performance of different generative models by Bloom’s Taxonomy. ChatGPT 3.5, ChatGPT 4o, and Gemini 2.5 answered 23 (76.7%), 27 (90%), and 28 (93.33%) correctly for questions in the Remember level, respectively. We also observed that the number of questions answered incorrectly reduced from ChatGPT 3.5 (6 questions) to ChatGPT 4o (3 questions) to Gemini 2.5 (2 questions). For the Understand level, ChatGPT 3.5 answered the least number of questions correctly (22 questions, or 73.3%), followed by Gemini 2.5 (25 questions, or 83.33%), and ChatGPT 4o (28 questions, or 93.33%). The number of questions answered incorrectly at the Understanding level increased (12 questions) compared to that at the Remember level (10 questions). In addition, we observed that ChatGPT 3.5 achieved the least accuracy in the Apply level, with 18 questions answered correctly, followed by Gemini 2.5 (24 questions answered correctly), and ChatGPT 4o (25 questions answered correctly). Finally, in the Analyze level, ChatGPT 3.5 was able to answer 15 questions correctly (50%), followed by both ChatGPT 4o and Gemini 2.5, where both answered 22 questions (73.33%) correctly. We observed that the number of questions answered incorrectly or partially correctly increased with the difficulty levels of Bloom’s Taxonomy.

**Figure 6:**
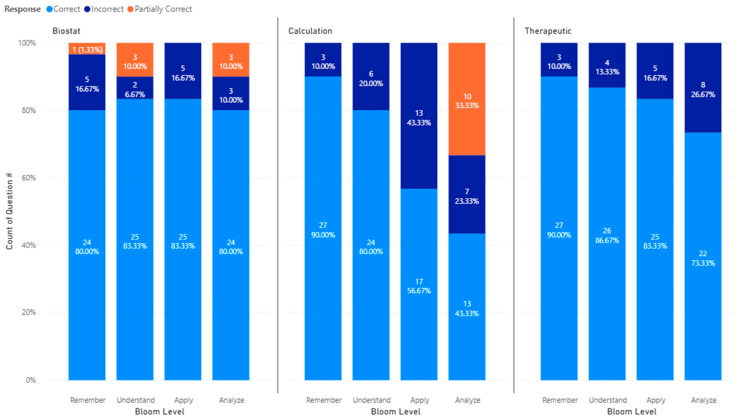
Accuracy performance by topics and Bloom’s Taxonomy levels.

Next, Figure 6 shows the accuracy performance of all models by topics and Bloom’s Taxonomy levels. For the biostatistics topic, we observed that the AI models’ performance was similar across all of Bloom’s Taxonomy levels, with 24 (80%) responses being correct, and 6 (20%) responses being partially correct or incorrect.

For calculation questions, we observed that the number of incorrect or partially correct responses increased from the Remember to the Apply level in Bloom’s Taxonomy. Particularly, there were only 3 (10%) incorrect responses in the Remember level, but it increased to 6 (20%) incorrect responses in the Understand, 13 (43.33%) in the Apply, and 17 (56.66%) in the Analyze level.

For therapeutic questions, we observed a similar trend with the number of incorrect responses increasing gradually from the Remember level to the Analyze level. There were 27 (90%) correct responses, and 3 (10.0%) were incorrect at the Remember level. For questions categorized as Understand, 26 (86.7%) were correct, and 4 (13.3%) were incorrect. Furthermore, for questions categorized as Apply, 25 (83.3%) were correct, and 5 (16.7%) were incorrect. Finally, for questions categorized as Analyze, 22 (73.3%) were correct, and 8 (26.7%) were incorrect. The accuracy decreased by one question (3.3%) from each level of Remember to Understand to Apply and decreased further by three questions (10%) from Apply to Analyze.

## 4. Discussion

### 4.1. Overall Accuracy and Reliability of ChatGPT in Pharmacy Education

The results of this study demonstrate that generative AI models exhibit strong overall accuracy in comprehending pharmacy-related content. Each AI model was evaluated using the original 120 questions, resulting in a total of 360 questions across all three models. The aggregate accuracy rate of 77.5% indicates that AI models can provide correct and educationally valuable responses to the majority of pharmacy-related queries across a broad spectrum of topics. This level of performance suggests that these models can serve as effective supplementary tools for pharmacy students, especially in reinforcing understanding of foundational and applied topics.

Notably, the progression from ChatGPT 3.5 to ChatGPT 4o reveals significant improvements in accuracy and depth of response. ChatGPT 4o consistently outperformed both ChatGPT 3.5 and Gemini 2.5 across all topic areas, achieving up to 97.5% accuracy in therapeutics questions. This trend can be explained by the ongoing model refinement and suggests that newer generations of AI models are increasingly capable of understanding domain-specific language and reasoning patterns in medical and pharmaceutical contexts.

### 4.2. Topic-Specific Performance and Educational Implications

A closer examination of topic-level performance reveals substantial variation in AI accuracy across different areas of pharmacy education. For therapeutics, ChatGPT 4o achieved remarkably high accuracy (97.5%), suggesting strong content knowledge and potential for relevance and application as a tool in pharmacy education. The model’s capacity to provide highly accurate and contextually appropriate responses indicates that it can serve as a valuable tutor-like resource for explaining drug mechanisms, therapeutic choices, and clinical guidelines.

In contrast, performance in pharmaceutical calculations was relatively lower (67.5% accuracy overall in ChatGPT 4o and 70% accuracy overall in Gemini 2.5). This finding indicates that while AI models can handle conceptual reasoning well, they remain more prone to errors when required to perform precise quantitative reasoning or multi-step calculations. Such results are consistent with the limitations of large language models, which rely on probabilistic pattern recognition rather than deterministic computation. Similar findings were reported by Cornelison et al. [13] when utilizing ChatGPT to evaluate the accuracy of large language models in pharmaceutical calculations. While pharmaceutical calculations are considered a crucial component of pharmacy education, there is limited evidence supporting specific interventions or assessments [14]. Consequently, the role of AI to support pharmaceutical calculations may be considered. Therefore, when used in pharmacy education, AI models should be considered a complement but not a replacement for structured practice in dosage calculations, pharmacokinetic modeling, and statistical computations. Students and educators should be aware of this limitation and use AI-assisted responses as a conceptual scaffold rather than a definitive source for numerical accuracy.

Performance in biostatistics at 81.7% accuracy overall suggests that AI models can effectively aid students in interpreting research data, understanding *p*-values and confidence intervals, and applying statistical concepts to pharmacoeconomic or clinical study evaluations. This potential can be particularly advantageous in capstone or research courses where students engage in literature reviews and data analysis.

### 4.3. Performance Across Bloom’s Taxonomy

The analysis by Bloom’s Taxonomy provides deeper insight into how AI models support cognitive skill development. The highest accuracy rates were observed at the Remember (86.7%) and Understand (83.3%) levels, reflecting ChatGPT’s strength in factual recall and conceptual explanation. This aligns with its design as a language-based model trained in vast text corpora, making it adept at retrieving, summarizing, and explaining established knowledge. Consequently, the AI models studied can serve as a highly effective tool for reviewing factual material, such as drug classifications, mechanisms of action, or regulatory frameworks.

However, accuracy declined notably at higher cognitive levels: Apply (74.4%) and Analyze (65.6%). This pattern suggests that while ChatGPT can articulate knowledge effectively, its ability to apply principles to novel scenarios, such as complex case analyses or pharmacotherapy decision-making, remains limited. The increased number of incorrect or partially correct answers at these higher levels indicates that true clinical reasoning and analytical synthesis still require human judgment and contextual understanding. Educators should therefore frame AI-based learning activities as opportunities for guided reasoning rather than autonomous clinical decision-making.

### 4.4. Comparative Model Analysis and Evolution of Capabilities

When comparing individual models, ChatGPT 4o consistently demonstrated the highest performance across all content areas and cognitive levels. It showed advancements in reasoning accuracy, particularly in higher-order questions (e.g., Analyze-level questions). Gemini 2.5 also performed well, although slightly below ChatGPT 4o, indicating that competition among AI model developers continues to drive improvements in domain-specific understanding. The incremental accuracy from ChatGPT 3.5 to 4o reinforces the pedagogical importance of using the latest AI versions for educational support, as they incorporate broader contextual comprehension and more sophisticated reasoning frameworks.

### 4.5. Pedagogical Applications in Pharmacy Education

The implications of these findings are substantial for integrating AI-based tools into pharmacy education and add to the current body of knowledge. Many areas of pharmacy education have begun exploring the role of AI as a potential tool in pharmacy education. AI models can serve as an interactive tool for self-assessment and active learning, simulating a question-and-answer tutor that allows students to test their knowledge in areas such as therapeutics, pharmacology, and biostatistics while receiving immediate feedback. This self-directed approach encourages active recall and knowledge retention, enabling students to engage more deeply with the material. Our study found a decrease in accuracy with increasing complexity when answering exam questions, consistent with Edwards et al. [15] when comparing GPT-3.5 to pharmacy students on a therapeutics examination. This trend was also found by Shultz et al. [16], where GPT-4 favored generating exam questions of lower complexity. A similar study utilizing GPT-3.5 also suggests that AI can be beneficial as an education tool [17]. Consistent with our findings, AI may struggle with complex tasks, as Do et al. [18] reported when comparing AI performance on pharmacy skills assignments.

As the capability of AI improves, as shown in our study with improved performance from GPT 3.5 to GPT 4o, AI’s strong performance in therapeutics can make it particularly useful for augmenting case-based learning, where students can explore clinical scenarios, compare treatment options, and examine evidence-based recommendations. Educators have explored this use case for topics including HPV-associated oropharyngeal cancer [19] and sleep medicine [20], as well as drug dosing activities in pharmacokinetics [11]. Educators may consider incorporating AI models into hybrid exercises that prompt students to critically evaluate their responses for accuracy and completeness, thereby fostering analytical and reflective thinking.

Furthermore, the model’s high accuracy in biostatistics supports its use in teaching and research contexts, helping students grasp statistical concepts, interpret data outputs, and understand research methodologies from published studies. The AI models also offer considerable value in exam preparation and review, as their strength at lower Bloom taxonomy levels enables students to efficiently review factual and conceptual content, complementing traditional study materials and textbooks. Beyond student use, faculty members can benefit from the AI models’ capabilities as well. Instructors can use the model to draft, refine, or evaluate exam questions that align with Bloom’s taxonomy, ensuring cognitive diversity in assessment design. Collectively, these applications demonstrate AI models’ potential to enrich both teaching and learning processes in pharmacy education by providing personalized support, enhancing engagement, and reinforcing conceptual understanding.

## 5. Limitations

Despite its promising accuracy, AI models’ lower performance in quantitative and higher-order cognitive tasks highlights the need for structured integration and critical evaluation. Overreliance without verification could propagate misconceptions, particularly in dosage calculations or advanced pharmacotherapy. We plan to utilize inferential statistical testing to assess the results in our future research. Additionally, the current study is limited to three key content areas, including calculations, biostatistics, and therapeutics, which may not fully represent the breadth of a pharmacy curriculum. Future research will expand to include other areas such as pharmacology, medicinal chemistry, pharmaceutics, pharmacy law, and other areas such as those required by the Accreditation Council for Pharmacy Education in the Standards 2025 Appendix 1: Required Elements of the Didactic Doctor of Pharmacy Curriculum [21]. It should also assess AI-assisted learning outcomes longitudinally, focusing not just on response accuracy but also on improvements in students’ critical reasoning, problem-solving, and confidence in clinical decision-making. Educators and users of AI tools also may experience model drift and dataset shift over time, complicating implementation and evaluation [22]. Finally, our future study will also consider other popular AI models (e.g., DeepSeek) to have a more comprehensive evaluation.

## 6. Conclusions

This study evaluated the accuracy and educational potential of generative AI models (ChatGPT 3.5, ChatGPT 4o, and Gemini 2.5) in answering pharmacy-related exam questions across three major content areas: biostatistics, calculations, and therapeutics. The findings revealed that all models demonstrated strong overall accuracy, with ChatGPT 4o showing the most consistent improvement across topics and cognitive levels. The highest performance was observed in therapeutics, followed by biostatistics, while calculation-based questions posed the greatest challenge for all models. When analyzed through the lens of Bloom’s Taxonomy, AI models excelled at lower cognitive levels, such as remembering and understanding, but exhibited declining accuracy in higher-order tasks requiring application and analysis. These results suggest that AI models like ChatGPT can effectively reinforce foundational and conceptual learning but should be supplemented with instructor guidance for complex problem-solving and clinical reasoning. In addition, the findings provide timely insights into how generative AI can be integrated responsibly into pharmacy curricula to enhance self-directed learning, conceptual mastery, and evidence-based decision-making while maintaining academic rigor and professional standards.

Furthermore, this study highlights the growing capability of AI tools to serve as interactive learning companions, enabling pharmacy students to engage in self-directed study, case-based learning, and exam preparation. Faculty can also leverage these tools to enhance assessment design and instructional support. However, given the limited scope of topics and the observed variability in quantitative reasoning, the findings also indicate the importance of critical evaluation and responsible integration of AI into pharmacy curricula. Expanding future studies to additional domains such as pharmacology, medicinal chemistry, pharmaceutics, and pharmacy law, and assessing longitudinal learning outcomes, will provide a more comprehensive understanding of how AI can shape the development of competent, analytical, and confident pharmacy professionals.

## Figures and Tables

**Table 1:** Outcome definitions, example assessment questions, AI-generated responses, and evaluative comments used to classify generative AI outputs as Correct, Incorrect, or Partially Correct.

Outcome	Definition	Domain	Bloom’s Taxonomy Level	Examples	AI Model—Response	Comments
Correct	Generative AI selected or provided the correct answer(s).	Biostatistics	Remember	In a clinical trial, the investigator tried to disprove “there is no difference between treatment and placebo groups”. What was the investigator trying to disprove? (A) Null hypothesis (B) Alternative hypothesis (C) Type I error assumption (D) Confidence interval condition	Gemini 2.5: The investigator was trying to disprove the “null hypothesis,” which in this context states that there is no difference between the treatment and placebo groups. In clinical trials, the null hypothesis typically posits that there is no significant effect of the treatment being tested when compared to a placebo, implying that any observed differences in outcomes between the two groups are due to chance rather than the efficacy of the treatment. By attempting to disprove this hypothesis, the investigator aims to provide evidence supporting the “alternative hypothesis,” which suggests that there is a significant difference between the treatment and placebo groups, indicating that the treatment has a specific effect. This is a fundamental approach in hypothesis testing used to evaluate the effectiveness of treatments in clinical research. Therefore, the correct answer is A.”	The AI model elaborated in detail on its rationale and made the correct choice for the final answer (option A.) As a result, we classified the answer as “Correct.”
Incorrect	Generative AI selected or provided an incorrect answer(s).	Therapeutic	Apply	A 47-year-old male comes to the clinic for routine follow-up. He has hypertension and is a smoker. His 10 Year ASCVD risk is 8.5%. Which of the following would be the best treatment recommendations according to the ACC/AHA Blood Cholesterol guidelines? (A) Initiate simvastatin 20 mg daily (B) Initiate gemfibrozil 600 mg twice daily (C) Initiate atorvastatin 40 mg daily (D) Nonpharmacotherapy only, and continue to monitor	ChatGPT 3.5: According to the ACC/AHA Blood Cholesterol guidelines, the primary consideration for statin therapy is the patient’s 10-year atherosclerotic cardiovascular disease (ASCVD) risk. In this case, the patient’s 10-year ASCVD risk is 8.5%. The guideline recommendations for statin therapy are as follows: - Consider moderate-intensity statin therapy for individuals aged 40–75 years with diabetes and an LDL-C level of 70–189 mg/dL. - For individuals without diabetes aged 40–75 years with an LDL-C level of 70–189 mg/dL and an estimated 10-year ASCVD risk of 7.5% or higher, a moderate- to high-intensity statin is recommended. Therefore, the most appropriate option for this patient would be: C.	The rationale is fairly good, but the final choice it made was wrong. The correct answer should be A. In this case, we classified the output as “Incorrect.” At the time of this input, the major guideline was the 2018 AHA/ACC multi-society guideline on the management of blood cholesterol, which recommended that for primary prevention for adults 40–75 at an intermediate-risk (10-year ASCVD 7.5–19.9%) a moderate intensity statin should be recommended, not moderate-to high-intensity as specified in the AI output. Atorvastatin 40 mg daily (C) is considered high intensity, and simvastatin 20 mg (A) is considered moderate intensity.
Partially correct	For multiple-selection questions, generative AI selected one or more—but not all—correct options. For free-response questions, partial responses typically captured the right approach or some correct elements but missed a key constraint or detail (e.g., omitted an edge case, provided incomplete steps, or mixed correct facts with a minor inaccuracy).	Calculation	Analyze	Patient name: AB DOB: 20 December 2016 Rx: Cefixime oral suspension 100 mg/5 mL Sig: take 1 and 1/2 tsp PO daily Disp: 10-day supply Which of the following statements are true? Select ALL that apply. (A) This prescription is for a young boy. (B) The drug is taken 7.5 mL every day. (C) The dispensed amount should be at least 75 mL. (D) The prescription cannot be refilled.	ChatGPT 4o: “Given the information: - Cefixime oral suspension: 100 mg/5 mL - Sig: take 1 and 1/2 tsp PO daily - Disp: 10-day supply Let’s evaluate the statements: A. “This prescription is for a young boy.” - Based on the DOB provided (20 December 2016), and without knowing the current date, we can infer that the patient, AB, is relatively young. Given the typical age for using “young boy” to describe someone, this statement is likely true. B. “The drug is taken 7.5 mL every day.” - 1 teaspoon (tsp) is approximately 5 mL, so 1 and 1/2 tsp would be 7.5 mL. Therefore, this statement is true. C. “The dispensed amount should be at least 75 mL.” - If the drug is taken at 7.5 mL every day and the prescription is for a 10-day supply, the total volume needed would be 7.5 mL/day * 10 days = 75 mL. Therefore, this statement is true. D. “The prescription cannot be refilled.” - Without additional information regarding the prescription rules or regulations specific to medication or jurisdiction, this statement cannot be determined as true or false based solely on the information given. Prescriptions for antibiotics typically have a defined course and may not need a refill if the treatment is meant to be completed within the dispensed amount. However, the statement’s validity regarding refills would depend on the prescribing doctor’s instructions or regulatory policies, which are not provided here. Based on the provided information, the true statements are as follows: - A. This prescription is for a young boy. - B. The drug is taken 7.5 mL every day. - C. The dispensed amount should be at least 75 mL. Statement D cannot be definitively assessed as true or false with the given details.	The AI model provided a very detailed explanation and rationale of the choices. However, the correct options are B and C. Due to A were selected as well by the AI model, in this case, we classified it as “Partially Correct”.

## Data Availability

The raw data supporting the conclusions of this article will be made available by the authors on request.
